# Management of eosinophil-associated inflammatory diseases: the importance of a multidisciplinary approach

**DOI:** 10.3389/fimmu.2023.1192284

**Published:** 2023-05-17

**Authors:** Santiago Quirce, Borja G. Cosío, Agustín España, Ricardo Blanco, Joaquim Mullol, Cecilio Santander, Victoria del Pozo

**Affiliations:** ^1^ Centro de Investigación Biomédica en Red de Enfermedades Respiratorias (CIBERES), Madrid, Spain; ^2^ Department of Allergology, Hospital Universitario La Paz, Instituto de Investigación Hospital Universitario La Paz (IdiPAZ), Madrid, Spain; ^3^ Department of Respiratory Medicine, Hospital Universitari Son Espases, Fundación Instituto de Investigación Sanitaria Islas Baleares (IdiSBa), Palma de Mallorca, Spain; ^4^ Department of Dermatology, Clínica Universidad de Navarra, Pamplona, Spain; ^5^ Department of Rheumatology, Hospital Universitario Marqués de Valdecilla, Immunology Group, Instituto de Investigación Sanitaria Marqués de Valdecilla (IDIVAL), Santander, Spain; ^6^ Rhinology Unit and Smell Clinic, Ear, Nose and Throat (ENT) Department, Hospital Clínic de Barcelona, Universitat de Barcelona (UB) - Instituto de Investigaciones Biomédicas August Pi i Sunyer (IDIBAPS), Barcelona, Spain; ^7^ Department of Gastroenterology and Hepatology, Hospital Universitario La Princesa, Centro de Investigación Biomédica en Red de Enfermedades Hepáticas y Digestivas (CIBEREHD), Instituto de Investigación Sanitaria del Hospital Universitario de La Princesa (IIS-IP), Madrid, Spain; ^8^ Universidad Autónoma de Madrid (UAM), Madrid, Spain; ^9^ Immunoallergy Laboratory, Immunology Department, Instituto de Investigación Sanitaria Fundación Jiménez Díaz (IIS-FJD), Madrid, Spain

**Keywords:** eosinophil, eosinophilic inflammation, eosinophil-associated disease, multidisciplinary management, expert opinion

## Abstract

Elevated eosinophil counts in blood and tissue are a feature of many pathological processes. Eosinophils can migrate and accumulate in a wide variety of tissues and, by infiltrating a target organ, can mediate the development of several inflammatory diseases. The normalization of eosinophilia is a common biomarker of a treatable trait and can also be used as a prognostic and predictive biomarker since it implies a reduction in type 2 inflammation that contributes to disease pathogenesis. Biological therapies targeting this cell type and its proinflammatory mediators have been shown to be effective in the management of a number of eosinophilic diseases, and for this reason they constitute a potential common strategy in the treatment of patients with various multimorbidities that present with type 2 inflammation. Various biological options are available that could be used to simultaneously treat multiple target organs with a single drug, bearing in mind the need to offer personalized treatments under the umbrella of precision medicine in all patients with eosinophil-associated diseases (EADs). In addition to reviewing these issues, we also discuss a series of perspectives addressing the management of EAD patients from a multidisciplinary approach, with the collaboration of health professionals from different specialties who manage the different multimorbidities that frequently occur in these patients. We examine the basic principles of care that this multidisciplinary approach must cover and present a multidisciplinary expert opinion regarding the ideal management of patients with EADs, from diagnosis to therapeutic approach and follow-up.

## 1 Introduction: eosinophilic inflammation as a common ground in different diseases

Eosinophils make up about 3% of all circulating leukocytes in peripheral blood under homeostatic conditions (1). Their development in the bone marrow, activation, release into the bloodstream and survival in peripheral tissues depend on the orderly interaction of multiple transcription factors and cytokines (2–5), among which interleukin-5 (IL-5) is the most specific for this cell type (6, 7). IL-5 promotes the development and recruitment of eosinophils by acting synergistically with other molecules such as eotaxins (8), alarmins (thymic stromal lymphopoietin [TSLP]) and IL-4, IL-13, IL-25, IL-31 and IL-33, among others (9).

**Figure 1:**
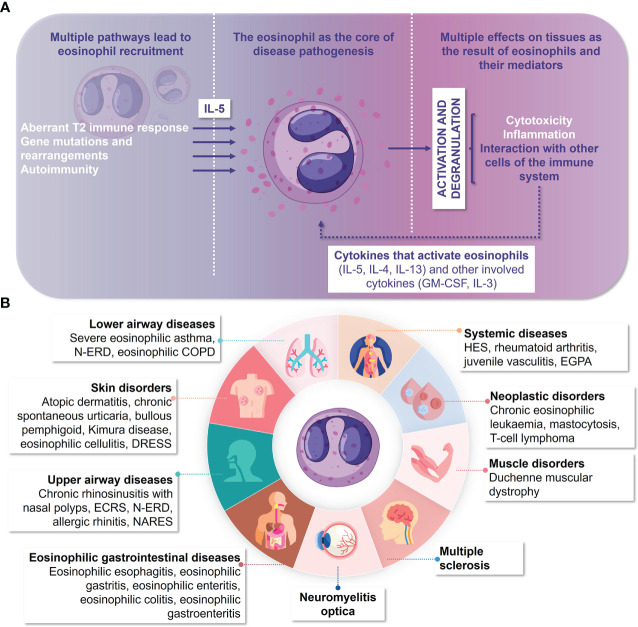
The eosinophil as the main cell responsible for diseases that present with eosinophilic inflammation. **(A)** The eosinophilic inflammation cycle. **(B)** Migration of eosinophils to the different tissues in which they cause eosinophilic diseases. COPD, chronic obstructive pulmonary disease; DRESS, drug reaction with eosinophilia and systemic symptoms; ECRS, eosinophilic chronic rhinosinusitis; EGPA, eosinophilic granulomatosis with polyangiitis; GM-CSF, granulocyte macrophage colony-stimulating factor; HES, hypereosinophilic syndrome; IL, interleukin; NARES, non-allergic rhinitis with eosinophilia syndrome; N-ERD, nonsteroidal anti-inflammatory drug-exacerbated respiratory disease; T2, type 2 immune response.

Eosinophil counts increase significantly in some pathological processes, both in blood and tissue compartments (10), mainly as consequence of a multi-immune response involving type 2 (T2) cytokines and other proinflammatory mediators. The active metabolism of eosinophils and the cytoplasmic secretory granules they contain allow them to act as inflammatory cells wherever they are recruited (11). Thus, they can trigger powerful cytotoxicity and inflammation processes that persist cyclically and chronically by interacting with other immune system cells that sustain their degranulation activity and lead to inflammation (12–18) (**Figure 1A**). The eosinophilic mediators-containing toxic granules are believed to damage the different tissues where these cells are specifically recruited. This suggests a direct causal relationship between eosinophils and the tissue damage that manifests in diseases such as asthma, chronic rhinosinusitis with nasal polyps (CRSwNP), eosinophilic gastrointestinal diseases (EGIDs) and systemic disorders as eosinophilic granulomatosis with polyangiitis (EGPA) and hypereosinophilic syndrome (HES), among others. All these supports the fact that eosinophil depletion is notably considered in the therapeutic interventions performed in all these pathologies (19–21). Besides, recent publications reported the involvement of T2 inflammation and eosinophils in the context of diverse autoimmune pathologies related to central nervous system, such as multiple sclerosis and neuromyelitis optica (NMO) (22, 23) (**Figure 1B**). As an example, the active role of eosinophils in the generation of tissue damage in the latter disease has been described in NMO mouse models in which the depletion of eosinophils by using an anti-IL-5 antibody was related to the reduction of tissue damage (24). Besides, the extent to which eosinophils participates in other pathologies such as Duchenne muscular dystrophy remains controversial (25).

While it is important to remark the existence of eosinophilia in the absence of Th2-related processes as in the case of neoplastic or idiopathic HES, in which neither a clonal nor a reactive underlying pathology is detected (26–28).

The identification of eosinophilia as a biomarker of a treatable trait, namely T2 inflammation, has been classically associated with the pathogenesis of a number of respiratory diseases, up to a point that eosinophilia has been used to guide the treatment of some of these diseases (29, 30). A threshold of 300 cells/μL has been postulated to be used when identifying patients candidate to receive a treatment based on inhaled corticosteroids, according to the Global Strategy for the Diagnosis, Management and Prevention of Chronic Obstructive Lung Disease (GOLD) (30). Other definitions of blood eosinophilia are those defined by and absolute eosinophil count of >0.5 × 10^9^/L, whereas hypereosinophilia relates to an absolute eosinophil count ≥ 1.5 × 10^9^/L (28, 31–37). Nonetheless, the normal range of blood eosinophil counts in general population has not been well established yet and this threshold has been criticised and interpreted as a normal eosinophil range (38, 39). Besides, not only eosinophil counts are important, but also the period of time during which eosinophilia is maintained. A difference between transient or episodic and persisting eosinophilia has also been defined (28). Clonal or neoplastic eosinophilia is always related to a persistent increase in the counts of these cells unless specific anti-neoplastic therapies are used to target it (28, 31–37). Also, eosinophil variability, as measured by the recently proposed Eosinophil Variability Index, is associated with the hospitalization of patients with asthma (40).

Eosinophils migrate to the respiratory mucosa and release a large quantity of mediators that trigger a characteristic T2 inflammatory process resulting in airway remodelling (41–43). The eosinophilic cationic protein (ECP) is one of the eosinophil-derived mediators most widely studied among the biomarkers of eosinophil activation (44–46). Although it seems to be unspecific for the diagnosis of eosinophil-associated respiratory diseases such as asthma, ECP is considered as a useful biomarker of airway inflammation and its abundance in peripheral blood has been associated to the control of asthma, and considered as a good biomarker to monitor the efficacy of classical and new biological therapies used in the treatment of this disease (44–47). This eosinophilic inflammation increases during respiratory exacerbations and impairs disease control (48). It also induces the onset of other respiratory diseases such as allergic rhinitis, non-allergic rhinitis with eosinophilia syndrome (NARES) and chronic rhinosinusitis (CRS) (49, 50). In one third of patients with asthma, CRS induces the development of nasal polyps (CRSwNP), which is associated with T2 inflammation in up to 87% of patients (50–55), especially in the presence of eosinophil-associated multimorbidities (56–58). In fact, several studies and Clinical Practice Guidelines define eosinophilia as one of the main criteria to define T2 inflammation, both when measured in peripheral blood and most importantly at the nasal polyp tissue level (50, 52, 59–61). Chronic obstructive pulmonary disease (COPD) and asthma-COPD overlap (ACO) syndrome are two highly prevalent respiratory entities that can also present this T2 signature; the involvement of eosinophils is also important for the pathogenesis (62–66) and treatment of these diseases (67). COPD is a heterogeneous condition characterized by a chronic airflow limitation, defined by a post-bronchodilator FEV1/FVC <0.7, with different components and mechanisms involved (68), while ACO defines a situation in which a persistent airflow limitation is associated with clinical features compatible both with asthma and COPD (69–71), and underlying eosinophilic airway inflammation may be present (62).

The eosinophil is also one of the cell types involved in several pathogenic immune responses that occur in the skin, and systemic involvement may also be a factor, given the interaction of this compartment with the peripheral lymph nodes (72–74). Atopic dermatitis (AD) is the dermatosis most commonly related to this cell type. It is caused by the development of dysregulated T2 immune responses that lead to eosinophil recruitment and activation (75, 76). Similarly, the eosinophil appears as the primary effector cell in the pathogenesis of bullous pemphigoid (BP), a disease typically classified as humoral (77). In fact, the presence of activated eosinophils along the basement membrane of the epidermis has been described in patients with BP (78–81). Furthermore, the interaction of eosinophils and immunoglobulin E (IgE), the production of which is mediated by T2 cytokines (IL-4, IL-5), amplifies the inflammatory response in this disease and the associated tissue damage (82). Likewise, eosinophils play a more important role than previously thought in skin conditions such as chronic spontaneous urticaria (CSU) (83), typically associated with mast cell activity (84). In CSU, the eosinophils accumulate together with these cells in the characteristic perivascular infiltrates of the disease (83, 85), which results in an inflammatory response that subsequently manifests as the typical hives associated with this condition (83).

As mentioned above, eosinophil-mediated inflammation can result in pathological processes at the systemic level. EGPA and HES are two of the systemic diseases in which eosinophils are recruited and activated in peripheral blood and various tissues, mediating proinflammatory effects that lead to widespread tissue destruction (86–89). The pathogenesis of EGPA is partly the result of the complex interaction between innate and adaptive immunity, involving not only eosinophils but also other cells (15, 90, 91). In fact, in the opinion of the American College of Rheumatology, peripheral blood eosinophilia is one of the clinical-pathological factors of interest in the diagnosis of EGPA, since eosinophils appear to be involved in some of its main complications, including pulmonary infiltrates, cardiomyopathy, gastrointestinal and respiratory manifestations, and axonal neuropathy (15, 92). Likewise, it has been postulated that the aetiology of HES is related to a primary dysregulation of eosinophil proliferation or a secondary dysfunction of the T2 immune response, as observed in virus infections or lymphoproliferative disorders (93, 94). Eosinophilic infiltration and local release of proinflammatory mediators have been linked to various HES-associated effects on the heart, skin, nervous system, lungs, liver, and gastrointestinal tract (93, 94).

Eosinophils usually occur in very low proportions throughout the gastrointestinal tissue, although their infiltration into these tissues gives rise to different types of clinical conditions, including EGIDs, depending on the specific location where the infiltration occurs (95, 96). EGIDs include, among others, eosinophilic esophagitis (EoE), and eosinophilic gastritis and gastroenteritis (95, 97). Eosinophils are the characteristic cells in EoE, as their density in the oesophageal epithelium is one of the main histological findings of the disease, and their disappearance determines the effectiveness of its treatment (98).

## 2 Treatment of eosinophil-associated diseases

Normalization of eosinophilia constitutes an important goal in various eosinophil-associated diseases (EADs), since it is a surrogate biomarker of reduction of the T2 inflammation that contributes to the pathogenesis of these conditions, leading to significant clinical improvements (99–104). Eosinophil counts can also be considered a potential inflammatory biomarker in these diseases (75, 76, 105–112) and a predictor of the response to some of the drugs used in their treatment, such as corticosteroids (65, 66, 99, 113–119).

### 2.1 Current advances

Treatment with biological drugs is recommended in patients with difficult-to-treat and severe adult-onset eosinophilic asthma (69, 103, 120, 121). Achieving a good control of the symptoms, minimizing future risk of asthma-related morbidity and mortality, exacerbations, persistent airflow limitations and treatment side effects are the main long-term goals of asthma (69). The use of biological therapies such as monoclonal antibodies has been described as useful in the treatment of this disease (122). In these patients, the use of anti-IL-5 drugs (mepolizumab, reslizumab), that bind circulating IL-5 (122), inhibits the maturation and differentiation of eosinophils, thereby decreasing their counts in peripheral blood and tissue (123–125). Furthermore, drugs targeting the IL-5 receptor, IL-5Rα, such as benralizumab, enhance the elimination of eosinophils and their precursors by antibody-dependent cellular cytotoxicity (124, 126), with a correlation between the initial eosinophil counts and the response to some of these treatments (127). Drugs targeting other mediators of eosinophil recruitment to the respiratory mucosa such as the epithelial alarmin TSLP (tezepelumab) have also been related with a specific reduction in the number of eosinophils in the airway submucosa, leading to a reduction in eosinophilic airway inflammation (122, 128) that seems to accompany the improvements in asthma clinical outcomes after the use of this drug (129). These drugs have been shown to be effective in controlling severe eosinophilic asthma, reducing the number of exacerbations, diminishing or eliminating the use of systemic corticosteroids (130–132), increasing lung function, promoting general symptom control, and generally improving patient quality of life (QoL) (133–135). Along with intranasal corticosteroids, these biological drugs have also been approved for use in patients with severe, uncontrolled CRSwNP who have undergone endoscopic sinus surgery (136–139). Other biological drugs such as dupilumab (140–143) and omalizumab (144–147) have also been approved in this indication. Dupilumab blocks the signalling pathways of IL-4 and IL-13 by binding to IL-4Rα, which they share in hematopoietic cells such as B cells, CD4^+^ helper T cells, and eosinophils (122), while omalizumab targets the Fc fragment of IgE and therefore reduces its levels in serum and inhibits its binding to its high-affinity receptor on mast cells and basophils (122). The use of the dupilumab results in the reduction of eosinophil migration and consequently in lung and blood eosinophil accumulation. This promotion of hypereosinophilia has been observed in 4-25% of patients treated with this drug, in whom the use of anti-IL-5 drugs is therefore preferable (148, 149).

Nevertheless, while these drugs reduce the eosinophil count in patients with CRSwNP, this does not appear to be the only mechanism responsible for their efficacy, as indicated by the results of the latest phase III trials (141, 145, 150). A phase IIa study suggested a response to drugs such as benralizumab and mepolizumab in some subgroups of patients with COPD and eosinophilia (151), but the results of phase III trials with these drugs did not support a significant reduction in exacerbations associated with this disease (152, 153).

Biological therapies targeting eosinophils and their mediators are also included in the therapeutic algorithm of some EGIDs, such as EoE (154). Dupilumab has been associated in this disease with a significant symptomatic, endoscopic and histological improvement in treated patients (155), while in eosinophilic gastroenteritis, a reduction in tissue eosinophilia has been observed in patients with this condition and concomitant HES treated with drugs such as benralizumab (156) and omalizumab (157). Blocking IL-13, a cytokine that activates and promotes eosinophil chemotaxis, also significantly reduces oesophageal eosinophilia and endoscopic and symptomatic disease activity (155). This has also been observed with biological drugs that target other molecules that are central to eosinophil activity in the oesophagus, such as Siglec-8 or eotaxins (155, 158).

The use of drugs directed against various eosinophilic therapeutic targets has also been postulated for some skin diseases associated with the action of this cell type. Suppression of the levels of some eosinophilic mediators and cytokines that regulate the development and activation of these cells has been shown to be effective in moderate forms of AD in paediatric patients (159). IL-31, another eosinophil-related cytokine (160), has been proposed as one of the most promising therapeutic targets in the treatment of AD, since some preliminary data on drugs targeting IL-31 suggest that they are effective in the management of pruritus, one of the symptoms that impacts heavily on the QoL of patients with AD (161). Other drugs that target T2 pathways, such as omalizumab or dupilumab, have shown an effective response in BP (162–165).

### 2.2 Future directions

With regard to future developments in the treatment of EADs, the efficacy of anti-IL-5/5Rα drugs is being studied in various lung diseases, such as allergic bronchopulmonary aspergillosis, eosinophilic pneumonia and bronchiectasis, and in other inflammatory and immune-mediated processes in which eosinophils may play an effector role (166).

Since eosinophils express low-affinity IgE receptors and their maturation and proliferation depend on IL-5, clinical trials in CSU are being conducted with drugs such as mepolizumab, reslizumab and benralizumab, given that many patients with this disease respond to treatments that inhibit IgE- or IL-5-mediated processes (167).

Resolution of the damage caused by eosinophil infiltration and the consequent release of toxic granules is another potential therapeutic approach in the treatment of systemic eosinophilic complications (115, 119, 168, 169). The normalization of eosinophil counts in peripheral blood and tissues is an important strategy in this regard, which is why the effects of different anti-IL-5/5Rα (reslizumab, benralizumab) and anti-IgE (omalizumab) therapies for the treatment of EGPA (170–172) and HES are being studied (173). Some of these, e.g., mepolizumab, are already approved for the treatment of both diseases (168, 174, 175).

Besides IL-5, some other factors have been related to prolonged eosinophil survival and promoted differentiation of these cells (176–180), and therefore must be considered as a plausible explanation of processes related to eosinophilia that are resistant to anti-IL-5 therapies. Some biological agents targeting IL-5 and other eosinophil-related cytokines (IL-4, IL-13) are also being evaluated for the treatment of a number of EGIDs, although in some cases the data from these studies are still preliminary and insufficient scientific evidence is available to recommend the use of these drugs in routine clinical practice (155, 156, 158, 181). In the context of EoE, the positioning of biological therapies is being evaluated to improve patient QoL and modify the natural course of the disease. These biological agents could be administered as part of the treatment algorithm in the management of patients refractory to corticosteroids, as maintenance therapy in corticosteroid-induced remission, or as treatment of patients with EoE and atopic multimorbidities (182, 183).

In summary, biological drugs that target the eosinophil and its mediators constitute a potential strategy in the treatment of patients with multimorbidities who present with T2 inflammation in which the eosinophil plays a fundamental role. The different therapeutic alternatives discussed, whether already in use or in development, could be useful for treating multiple diseases with a single drug, always with the aim of achieving personalized treatments and precision medicine in all patients with EADs.

## 3 Discussion: the multidisciplinary management of eosinophilic diseases

Eosinophilia is a common biomarker in several diseases involving T2 inflammation that require a comprehensive management approach involving specialists from different medical disciplines. This would ensure a holistic view that would address the basic principles of care needed in all EAD patients: prompt and accurate diagnosis; referral by a multidisciplinary team if necessary; access to safe and effective treatments; and greater awareness and education about EADs (184). All healthcare professionals responsible for treating these patients must understand these principles and implement them in their routine clinical practice. In fact, the Clinical Practice Guidelines focused on the management of some of the mentioned EADs have been already developed in the context of a multidisciplinary point of view based on the collaboration of several professionals from different Scientific Societies (59), and some approaches to develop multidisciplinary units for the management of some other EADs have been already published (185).

### 3.1 Prompt and accurate diagnosis of EADs and referral of patients by a multidisciplinary team

Patients with EADs should be diagnosed promptly and accurately (184). One of the main needs in this respect is to establish a multidisciplinary referral pathway for patients with EADs (184) that encompasses aspects ranging from the diagnosis of an EAD and its possible eosinophil-based multimorbidities to patient treatment and follow-up. This pathway does not necessarily require the implementation of multidisciplinary functional units, but it does require open two-way communication channels between the specialists who manage often concomitant eosinophilic multimorbidities. In any case, specialists who treat patients with any EAD should be aware of the coexistence of different eosinophilic conditions and as such, be prepared to consult with other specialists regarding the initial eosinophilic diagnosis and other signs or symptoms that the patient may present. It is important to emphasize that this awareness must be sustained over time, as recurrent eosinophilic multimorbidities can develop gradually, and therefore may be detected during follow-up and not necessarily at the time of initial EAD diagnosis.

**Figure 2:**
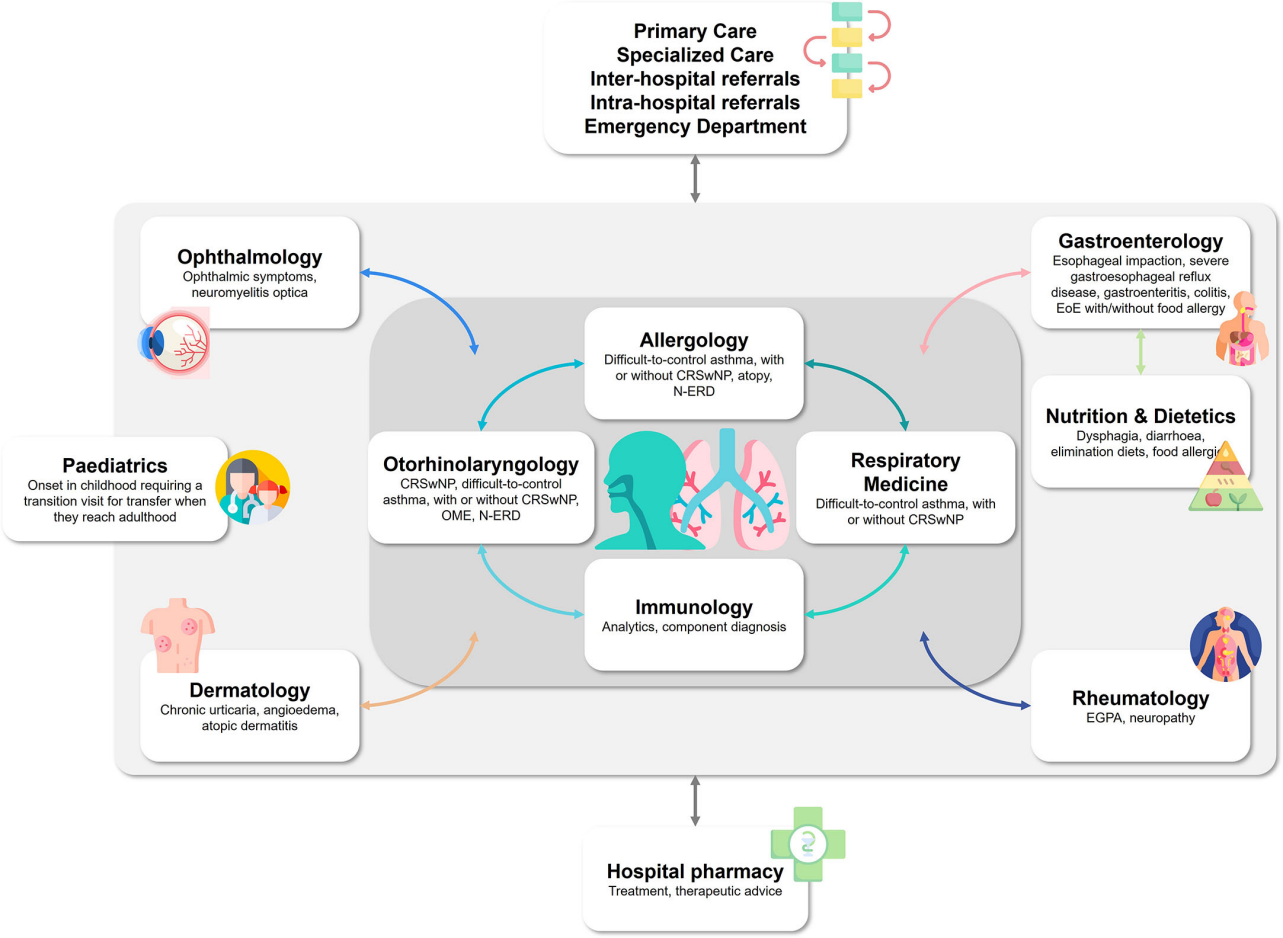
Perspectives on the ideal pathway for the multidisciplinary management of eosinophilic diseases. CRSwNP, chronic rhinosinusitis with nasal polyps; EGPA, eosinophilic granulomatosis with polyangiitis; EoE, eosinophilic esophagitis; N-ERD, nonsteroidal anti-inflammatory drug-exacerbated respiratory disease; OME, otitis media with effusion.

The advisability of multidisciplinary management depends on each patient’s profile: it may be unnecessary in some, while in others, due to the severity of their EADs, it may be crucial to detect and manage the onset of eosinophilic multimorbidities. Accordingly, referral criteria should be established to provide guidance when this is appropriate. Clinical practice experience tells us that a patient does not usually spontaneously notify the specialist of any signs or symptoms of a disease that they believe are not directly related to the reason for their visit, perhaps because they affect organs or systems that this particular specialist does not treat. Therefore, it may be very useful to establish a battery of basic structured interview questions that allow the clinician to probe the signs and symptoms of various eosinophilic multimorbidities. Each specialist should thoroughly examine the patient in order to detect the multimorbidities most frequently associated with their discipline, and maintain close contact with the specialists in the relevant specialties in each case (**Figure 2**). Criteria for interconsultation between specialists should be defined depending on the final objective of these interconsultations and on the availability of appropriate techniques for EAD diagnosis, treatment and follow-up, as already defined in the context of some of the most characterized EADs in which the intervention of medical professional from different specialties is needed (186–188).

### 3.2 Access to safe and effective therapies in the treatment of EADs

Patients with EADs should have access to safe and effective therapies (184), including specifically use of any of the different therapeutic alternatives available for the treatment of the multisystemic eosinophil-related manifestations that could occur concomitantly in a single individual. Beyond diagnosis, multidisciplinary management will ensure that a patient with eosinophilic multimorbidities can be treated with the best available therapeutic options. Particular consideration should be given to the biological therapies described above, the use of which is related with successful management of EADs, especially in patients with severe, difficult-to-treat EADs, in whom multiple different eosinophilic multimorbidities may occur. This is a scenario in which a personalized approach should prevail. Coordinated, comprehensive treatment of all manifestations of eosinophilic disorders using the appropriate therapy could provide great clinical benefits. Furthermore, it could potentially reduce the costs of care and treatment of these patients, who might otherwise receive different, possibly suboptimal interventions for each condition, if the aetiology common to each of the eosinophilic multimorbidities has not been properly analysed. Further considerations include indirect costs of care related to social and professional losses and a reduction in the QoL of inadequately treated patients.

### 3.3 Awareness and education about EADs

Awareness and education surrounding EADs should be strenuously promoted, especially among patients and the healthcare professionals who treat them (184). Both parties, and especially the latter, must also understand the benefits of multidisciplinary management. The patient must be the focal point in the management of the different multimorbid EADs and healthcare professionals involved in this management must accept the need, supported by scientific evidence, to collaborate with other specialists in certain cases, in order to achieve a holistic view of the patient with eosinophilic multimorbidities.

Healthcare professionals should also collaborate with patient associations to inform patients that they are entitled to multidisciplinary management and to ensure that they identify and report to the clinician any sign or symptom that could be associated with multimorbidities that present in association with the initially diagnosed EAD. Some of these diseases produce non-specific, long-term progressive symptoms to which the patient tends to adapt, resulting in many cases in delays before the patient seeks medical attention. In this respect, medical language will need to be adapted to facilitate patient awareness and understanding of the multidisciplinary pathway. Every patient with eosinophilic diseases should know that they may be treated by professionals from different medical areas who will be involved in the diagnosis and decision-making for optimal treatment of possible multimorbidities.

## 4 Conclusions

The evidence to date supports the idea that eosinophilia is a biomarker common to many diseases that present with T2 inflammation in different body systems. Biological therapies targeting eosinophils and their development, migration and activation in the tissues associated with the onset of EADs have led to a paradigm shift in the treatment of some of these diseases at the individual level. These drugs, therefore, could become, in the short-medium term, the preferred therapeutic approach in the simultaneous treatment of multimorbid diseases in which the eosinophil plays a pathogenic role. The interventions we describe here are simple and manageable in nature, but robust in effect. They will set us on a challenging path to a place where it will be possible to manage patients with EADs from a multidisciplinary perspective based on awareness and shared training in EADs and on collaboration between professionals from different medical specialties. There is little doubt that this approach will result in a significant benefit for patients with EADs.

## Data availability statement

The original contributions presented in the study are included in the article/supplementary material. Further inquiries can be directed to the corresponding author.

## Author contributions

All authors listed have made a substantial, direct, and intellectual contribution to the work and approved it for publication.
